# Investigating the intentions and reasons of senior high school students in registering for nursing education in China

**DOI:** 10.1186/s12912-023-01480-w

**Published:** 2023-09-12

**Authors:** Zhong Zhang, Chaoqun Yang, Ying Wang, Guoying Deng, Jian Chang

**Affiliations:** 1 Nursing Department, Shanghai General Hospital, Shanghai Jiao Tong University School of Nursing, Shanghai, 201620 China; 2 Shanghai Jiao Tong University School of Medicine, Shanghai, 200025 China; 3 Trauma Center, Shanghai General Hospital, Shanghai Jiao Tong University School of Medicine, Shanghai, 201620 China

**Keywords:** Nursing, Senior high school students, Education

## Abstract

**Background:**

A shortage of qualified nurses and their low level of educational qualifications hinders the development of global health services. Studies have proven the role of nursing education in addressing these problems. However, no related studies have focused on senior high school students in China. This study aimed to explore senior high school students’ intentions to learn nursing and identify the factors influencing their decision-making processes.

**Methods:**

An anonymous questionnaire was distributed to 8050 senior high school students, which included questions regarding their demographic characteristics, obtaining nursing specialty information, cognition of the nursing occupation and the impact of the COVID-19 on the nursing profession. Descriptive calculation, the chi-square test and logistic regression were used for the analysis.

**Results:**

Only 0.73% of the participants had a clear intention to study nursing. Academic performance and family support were significant predictors of students’ intentions to pursue nursing education. Students’ interest in nursing specialties was associated with their choice. There was a positive correlation between cognition of nursing occupation and students’ choice of nursing. Students’ experience of the COVID-19 pandemic also had a positive impact on their nursing career choice.

**Conclusion:**

This survey to some extent reflects the problem of nurses shortage in China. In addition, these findings may also provide a new perspective for predictors of nursing shortage and potential interventions.

**Supplementary Information:**

The online version contains supplementary material available at 10.1186/s12912-023-01480-w.

## Background

Nurses are a crucial aspect of the health care system and work in multidisciplinary teams to provide health care services to patients and society [1]. Since 2019, the widespread COVID-19 pandemic has further emphasized the necessity of nurses. However, the quantity and quality of the nursing workforce has suffered from a serious crisis. A model predicts that global demand for nursing workers will increase to 36 million workers, resulting in a worldwide shortage of 5.7 million nurses by 2030, which is more challenging in low-income and middle-income countries [1]. With China as an example, the ratio of nurses to the 1000 population is 2.3, compared with 12.9 in Germany by 2017 [2].

The nursing shortage will be aggravated due to the high turnover rate among the existing nursing workforce and ageing population. A recent study reported that 78.3% of nurses held a strong or extremely strong turnover intention in China, causing brain drain and shortage pressures [3]. By 2050, the population over 60 is estimated to rise to 478.9 million in China [4], leading to a major demand for nursing resources. Moreover, with the rapid growth of residents’ demand for high-quality health and the continuous development of ageing, traditional nursing models can no longer meet the current health needs. Nursing services extend from institutions to communities and families, and nursing staff should provide comprehensive nursing services such as professional care, health management, psychological care, rehabilitation promotion, and palliative care.

With rapidly advancing health care settings, there is a significant nursing demand in advanced care, elderly care, home care, and chronic disease health care management. Therefore, the shortage of well-prepared nurses not only threatens the health workforce system but also influences the quality of treatment. New nursing models such as advanced practice nurses (APNs) have set more requirements for nurses. An APN is defined by the International Council for Nurses (ICN) as a registered nurse with profound specialized knowledge, decision-making ability in complex problems, and expanded clinical practice skills. Furthermore, the definition of an APN from The American Nurse Association (ANA) is one who has achieved at minimum a graduate degree. The APN should also have the ability to diagnose and handle existing or potential health issues for individuals, families, and communities and then proceed to make clinical decisions on health issues. However, APNs are mainly aimed at cultivating high nursing talent, and the overall nursing construction of the nursing team is still an urgent problem to be solved.

Therefore, an increasing number of studies have highlighted the role of nursing education in addressing nursing shortage concerns [5]. In recent years, scholars worldwide have carried out abundant studies, and governments have developed various interventions to develop nursing education. However, fewer studies have focused on the source of future nurses. Based on China’s initial nursing education system, which includes three educational programs: the 3-year technical school for junior high school graduates, the college and the 4-year bachelor’s degree program for senior high school graduates, senior high school students are the main sources of registered nurses.

To fundamentally solve this dilemma, further investigations should concentrate on the career choices of high school students. Theories argue that the individual’s career choice is based on the perceived advantage or disadvantage of multiple factors associated with nursing, such as academic performance, financial level, working hours and volume of work [6]. Additionally, previous studies indicated that the motivation to pursue nursing might be employer factors, social factors, family factors, and personal factors [7, 8]. Meanwhile, the COVID-19 has led to an extraordinary amount of pressure on nursing staff, which might influence students’ understanding of nursing occupations [9].

In terms of social factors, a survey on the influencing factors of professional attitude shows that low social status is the most influential factor [10]. The societal perception of the nursing industry and the social status of the nursing profession can lead to a low level of professional enthusiasm among senior high school students before entering university, resulting in a lower number of senior high school students pursuing the nursing profession. Some researchers also believe that external factors in the market environment are also important factors that affect senior high school students’ choice of nursing majors, such as unreasonable employment policies and imbalanced development of nursing majors [10].

Employer factors may include personal development opportunities, salary benefits and job stability. According to a survey, personal development opportunities are considered as an important factor affecting senior high school students’ choice of nursing major [11]. Some researchers believe that Chinese medical institution managers have insufficient investment in training high-level nursing talent, resulting in no long-term training plan and promotion mechanism for the rational use of talent. Generally, nurses only have promotion opportunities for head-nurses [10]. The current management system for nursing staff places too much emphasis on work experience and does not take into account educational qualifications or potential development issues, which cannot reflect the value and advantages of higher education, making it difficult for highly educated nursing staff to fully utilize their talents, and high-quality talents are less likely to choose the nursing industry without seeing the prospects for personal career development [12]. Moreover, a study in Taiwan has shown that salary and benefits are also important factors to consider when making career decisions, especially for men. In Japan, it also showed that in the early 1990s, Japanese men joined the nursing industry mainly because they could not find alternative jobs after the economic bubble burst [13].

The family situation is also one of the important factors affecting senior high school students’ application for nursing majors. The influence of families on senior high school students’ willingness to apply mainly depends on the level of parental support for their children’s application for nursing majors, as senior high school students’ professional values may not yet built. Research shows that the greater the support from families for high school students to pursue nursing careers in the future, the greater their tendency to apply for nursing majors [14].

Professional values might also affect senior high school students’ willingness to pursue majors [15]. This may be influenced by the current market economy values, senior high school students placing more emphasis on social status, unit prospects, etc. For today’s Chinese society, it is generally believed that doctors have a high social reputation. For different patients, clinical doctors need to make independent judgements and provide personalized treatment strategies [16]. However, in China, nurses usually play the role of executors. Compared to clinical medicine majors, the low autonomy and low challenge of the nursing industry do not meet the professional expectations of high school students.

In summary, whether senior high school students will choose to pursue nursing majors may be influenced by many factors. With the acceleration of China’s ageing society, the demand for nursing staff has further increased. As a reserve force for talent cultivation, exploring the reasons for career choices for senior high school students’ willingness is beneficial for the shortage of nursing human resources. In addition, after the COVID-19 epidemic, society’s attention to the medical industry may have changed, which might also become one of the influencing factors. Therefore, this study systematically explored the current situation of senior high school students’ intention to learn nursing and identified characteristics of those with a clear intention to learn nursing and its associated factors. The study assessed the role of various covariates influencing students’ urge to pursue nursing, including individual factors, family background, obtaining nursing specialty information, cognition of the nursing occupation and the impact of the COVID-19 on the nursing profession. Findings from this study are essential in enhancing the understanding of nursing career options for high school students. Meanwhile, the study provides crucial evidence for nursing educators, nursing management, and policy-makers to obtain potential intervention strategies for student recruitment.

## Methods

### Participants and procedures

The cross-sectional design was conducted from March to April 2022. The study utilized convenience sampling and a cluster sampling method to recruit participants into the study. Based on convenience factors, we chose the four provinces for convenience sampling, including Zhejiang, Guangdong, Anhui and Sichuan provinces. Two, one and one of these provinces are located in eastern, middle and western China, respectively. Five senior high schools were randomly selected based on four provinces. In these five senior high schools, we applied cluster sampling method to select several classes, with all senior high school students in each class as the research object. Based on previous studies [11], we calculated the sample size with a 99% confidence level and a desired relative precision level of 0.04. Considering a 20% non-response rate, we appropriately increased the sample size based on this statistical calculation. A total of 9574 senior high school students were invited to participate in the study, with 37.0%, 43.6%, and 19.5% of them in senior one, senior two and senior three, respectively. The inclusion criteria were as follows: (1) enrolled in high school for more than six months (2) provided informed consent and volunteered to complete the questionnaires. The study utilized offline cluster sampling to distribute questionnaires. At each of these senior high schools, trained researchers thoroughly explained the purpose, completion process and voluntary nature of the study. Students were informed that all information would remain confidential. After ensuring the informed consent of students, hard copies of the questionnaire were distributed to them and collected anonymously.

### Data collection

**Fig. 1:**
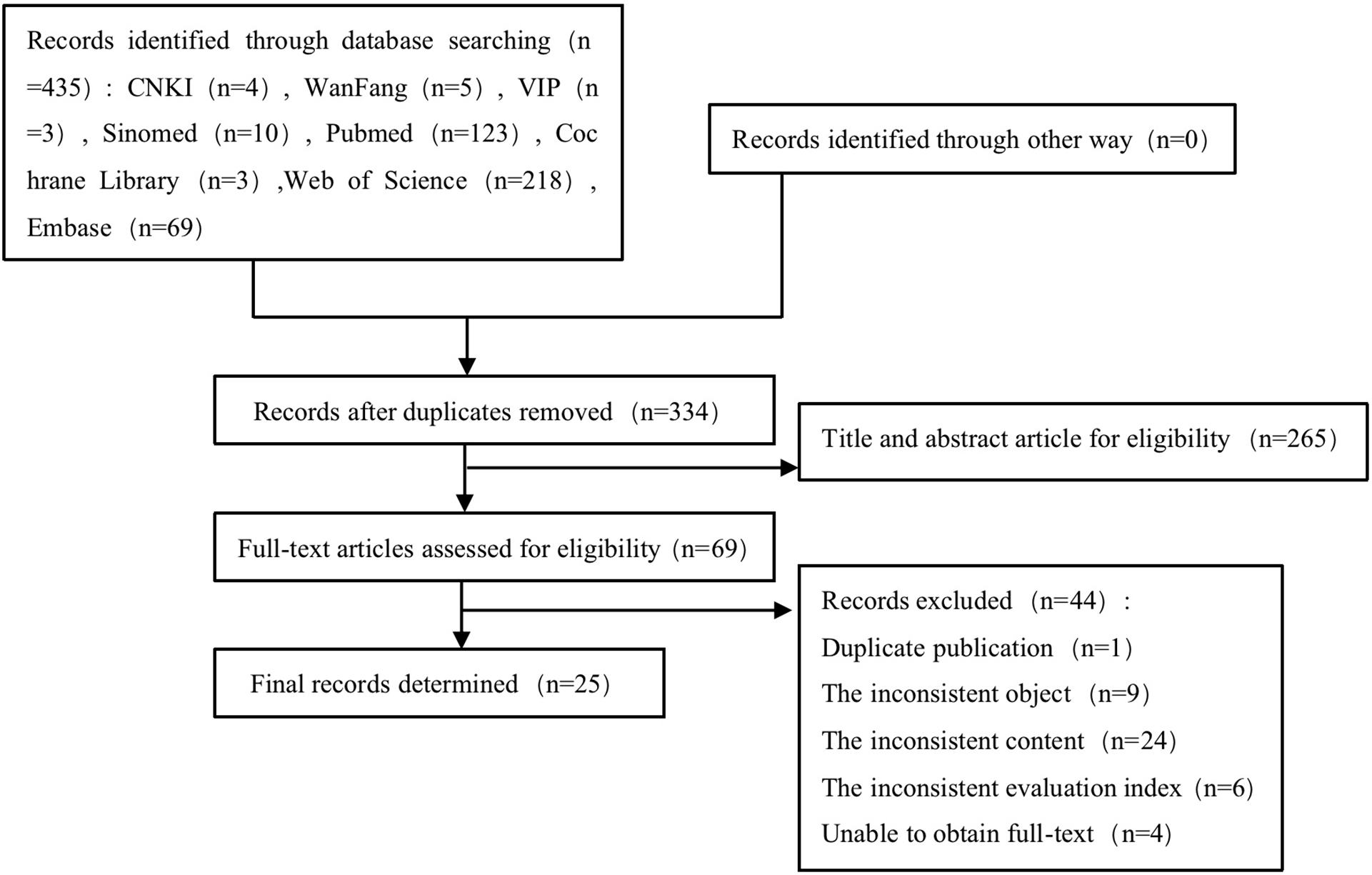
Search flow diagram

The study utilized a predefined questionnaire to collect data from the participants (the questionnaire can be downloaded in supplementary material). The entries in the questionnaire mainly come from literature review. The literature search was conducted both in Chinese and English databases, Fig. 1. The review covered a period from the database establishment to September 2021. Keywods included ‘senior school student*’, ‘high school student*’, ‘nursing’、‘career’, ‘nursing specialties’, ‘nursing specialty’, ‘Specialty, Nursing’, ‘Specialties Nursing’, ‘nursing major’ and ‘nursing profession’. Twenty-five literatures were determined after evaluation. Furthermore, the team consulted over twenty nursing educators by using Delphi method, they reviewed the study and research process to proposed some suggestions for the questionnaire. At last, two researchers with expertise in medical education from Shanghai General Hospital ascertain the reliability and validity of the questionnaire. A pilot study was conducted online with 200 senior high school students. The pilot study generated crucial insights into various aspects of data collection and enhanced revisions of the initial questionnaire. The final questionnaire consisted of 24 items (14 fixed response questions and 10 five-point Likert scale items), with a Cronbach’s alpha coefficient of 0.644. The Cronbach’s alpha coefficient values of fifteen nursing-related items and four COVID-19 epidemic-related items were 0.776 and 0.70, respectively.

The final questionnaire consisted of four parts. The first part of the questionnaire aimed to collect participants’ demographic information. The second part focused on students’ obtaining nursing specialty information. The third part assessed students’ cognition of the nursing occupation. The final part of the questionnaire assessed students’ experiences during the pandemic.

### Data analysis

The researcher used the Statistical Package for Social Sciences software (SPSS Inc., Chicago, IL, USA) to analyse the data. The study defined students selecting “I definitely will” in the questionnaire as having a clear intention to study nursing and those choosing “Maybe,” “I definitely won’t,” and “I have no idea” as having no apparent intention of pursuing a career in nursing. The researcher then compared students with or without clear intentions of pursuing a nursing career using inferential statistical methods. The study used percentages and frequencies to summarize factor variables and means and medians for continuous data. The study utilized univariate regression tests to generate crude estimates of the association between students’ intentions to pursue nursing education and the predictors of interest. Multiple regression analyses enhanced the generation of adjusted odds ratios to assess the significance of the interactions between potential covariates influencing students’ interest in pursuing a career in nursing. The study considered *p* < 0.005 as statistically significant for all inferential analyses.

### Ethical considerations

The study was approved by the Ethics Committee of Shanghai General Hospital because the survey was anonymous and did not include sensitive questions. Before completing the questionnaire, the respondents confirmed that they thoroughly understood the precautions. Participation in the research was voluntary, and all participants provided informed consent.

## Results

### Sample characteristics

**Fig. 2:**
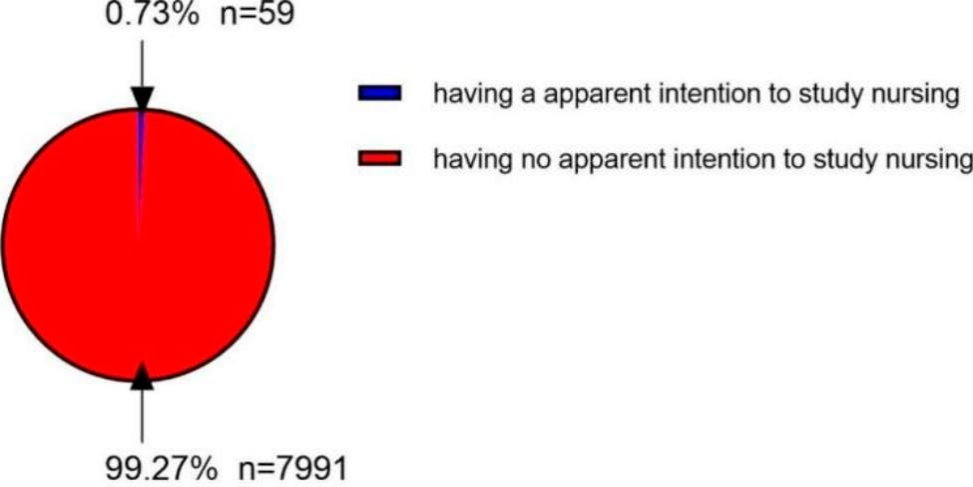
Students’ Intention to study nursing

A total of 9574 participants responded to the survey, yielding an overall response rate of 84.1% (8050/9574). Approximately 55.5% of the participants were female and 44.5% were male. Students in senior one, senior two and senior three accounted for 37.0%, 43.6%, and 19.5%, respectively. Among the respondents, only 0.73% had a clear intention of enrolling in nursing education (Fig. 2).

### Personal and family information

Personal information consisted of three variables: gender, preferred course and academic performance ranking. In terms of gender and preferred course, there was no statistical significance. Among the students ranked 71-94%, those with clear intentions of pursuing nursing education accounted for 2.12% (23/1084), higher than the other group. This result was statistically significant. (*p* < 0.001). (Table 1)

**Table 1:** Personal and Family Information

Item	N	N^*^(%)	χ^2^	P value
**Gender**			0.59	0.44
Male	3558	29(0.82)		
Female	4492	30(0.67)		
**Performance ranking**			30.85	< 0.001
Top 5%	634	3(0.47)		
6 − 30%	2351	19(0.81)		
31 − 70%	3720	12(0.32)		
71 − 94%	1084	23(2.12)		
Bottom 5%	261	2(0.77)		
**Course selection**			0.84	0.36
Liberal Arts (Partial Liberal Arts) Science (Partial Science)	2489 5561	15(0.6) 44(0.79)		
**Monthly income per person in family (RMB)**			3.239	0.52
< 2000	686	5(0.73)		
≥ 200, < 5000	3228	24(0.74)		
≥ 5000, < 10,000	2871	25(0.87)		
≥ 10,000, < 20,000	1068	5(0.47)		
≥ 20,000	197	0(0)		
One or both parents are medical workers*			1.737	0.188
Yes	542	7(1.29)		
No	7508	52(0.69)		
**Family attitude towards learning nursing**			53.022	< 0.001
Oppose	912	7(0.77)		
Neutral	5329	16(0.3)		
Support	1809	36(1.99)		

Abbreviations: medical workers*, People who are doctors, nurses, some sort of technician at the hospital, basic medical researchers, public health and prevention related workers, pharmacologists, etc.

Family information consisted of income, parents’ occupation, and family attitude towards further education in nursing. A total of 0.87% of participants from families with a monthly income per person of 5000–10,000 RMB and 1.29% of participants with one or both parents working in a medical institution expressed an apparent intention to pursue nursing education. However, no significant differences were found in these aspects. Family support was a significant predictor of students’ intentions to pursue nursing education. The proportion of students intending to pursue nursing education with support from parents was higher (1.99%) compared to those with opposing parents (0.77%) (*p* < 0.001). (Table 1)

### Obtaining nursing specialty information

Students’ understanding of the nursing specialty consisted of four variables: interest in nursing, the way to learn nursing-related information, frequency of receiving nursing-related information, and experience participating in nursing-related activities. A significant positive association was found between students’ interest in the nursing specialty and their clear intention to pursue nursing education (OR = 2.042, *p* < 0.001). Approximately 3.71% of 458 participants strongly interested in medicine had a clear intention to study nursing compared to the uninterested 0.34% of 1172. Multivariate analysis found that personal interest was a significant predictor of students’ interest in studying nursing (OR = 1.85, *p* < 0.001, Table 2).

**Table 2:** Obtaining Nursing Specialty Information

Item	N	N^*^(%)	Univariate regression		Multivariate regression	
			OR (95% CI)	P value	OR (95% CI)	P value
**Interest in nursing**			2.04(1.60, 2.61)	< 0.001	1.85(1.43, 2.40)	< 0.001
Totally uninterested	1203	8(0.67)				
Uninterested	1772	6(0.34)				
Neutral	3651	11(0.3)				
Interested	966	17(1.76)				
Very interested	458	17(3.71)				
**How related information is picked up**			0.96(0.78, 1.18)	0.68	1.08(0.86, 1.35)	0.51
Nothing	410	7(1.71)				
Traditional media (newspapers and magazines)	3157	23(0.73)				
New media (the Internet and APP)	710	1(0.14)				
School lecture	2337	14(0.6)				
People around	1436	14(0.97)				
**Frequency of receiving related information**			1.51(1.16, 1.91)	0.002	1.20(0.92, 1.57)	0.176
Never	1610	12(0.75)				
Extremely low	2400	13(0.54)				
Low	2909	12(0.41)				
High	1053	16(1.52)				
Extremely high	78	6(7.7)				
**Participation in related activities**			0.44(0.26, 0.74)	0.002	0.64(0.38, 1.09)	0.101
Yes	2195	27(1.23)				
No	5855	32(0.55)				

Abbreviations: N, total participants, N^*^, Number of students with a clear intention to pursue nursing education and career; OR, odds ratio; CI, confidence interval

Participants received nursing-related information mainly through traditional media (39.22%) and school lectures (29%). Students’ frequency of receiving nursing-related information was a significant predictor of students’ intention to study nursing (OR = 1.51 95%, *p* < 0.002). However, only 0.97% of the participants reported an extremely high frequency of receiving nursing-related information. Approximately 1.23% of the participants expressing interest in pursuing nursing education had participated in relevant nursing activities compared to 0.55% of participants who had not participated in nursing-related activities. Students’ participation in nursing-related activities was a predictor of their preference for pursuing nursing education. (OR = 0.44, *p* = 0.002). (Table 2)

### Knowledge of the nursing occupation

Participants’ cognition of the nursing occupation entailed students’ opinions on the employment situation of nurses, career prospects, nurses’ work environment, social status, and achievements. Students had different views on the employment situation of nurses, with 37.72% supporting job supply far less than demand and 33.81% supporting job supply far greater than demand. A total of 45.51% of participants were optimistic about nurses’ career prospects, 45.3% of those considered the nurses’ work environment to be friendly, 38.45% of those thought the social status of nurses was high, and 48.65% of those thought that nurses had a high sense of achievement. However, only 29.88% of participants knew about the work of nurses. (Table 3)

**Table 3:** Students’ Cognition of Nurse Occupation

Item	N	N^*^(%)	Univariate regression		Multivariate regression		
			OR (95% CI)	P value	OR (95% CI)	P value	
**Employment situation for nurses**			0.72(0.59, 0.88)	0.02	0.76(0.60, 0.96)	0.02	
Job supply far greater than demand	2722	27(0.34)					
Job supply and demand balance	2291	14(0.61)					
Job supply far less than demand	3037	18(0.38)					
**Nurse’s career prospects**			1.40(1.01, 1.95)	0.04	1.17(0.78, 1.77)	0.44	
Very pessimistic	159	4(2.52)					
Pessimistic	576	7(1.22)					
Neutral	3651	11(0.3)					
Optimistic	3081	22(0.71)					
Very optimistic	583	15(2.57)					
**Medical work environment for nurses**			1.43(1.07, 1.93)	0.02	1.34(0.88, 2.03)	0.17	
Extremely unfriendly	160	5(3.13)					
Unfriendly	920	0(0)					
Average	3329	21(0.63)					
Friendly	2808	16(0.57)					
Very friendly	833	17(2.04)					
**Social status**			1.37(1.04, 1.80)	0.03	0.56(0.36, 0.86)	0.01	
Very low	123	5(4.07)					
Low	1045	10(0.96)					
Average	3819	15(0.39)					
High	2435	16(0.66)					
Very high	628	13(2.07)					
**Sense of achievement**			1.493(1.10, 2.02)	0.01	1.51(1.00, 2.27)	0.048	
Very low	113	3(2.65)					
Low	794	6(0.76)					
Average	3227	10(0.31)					
High	2938	22(0.75)					
Very high	978	18(1.84)					
**Know the work of a nurse very well**			1.85(1.38, 2.49)	< 0.001	1.76(1.25, 2.49)	0.001	
Strongly disagree	372	1(0.27)					
Disagree	1196	8(0.67)					
Neutral	4077	17(0.42)					
Agree	1924	21(1.09)					
Strongly agree	481	12(2.49)					

Analyses showed a significant association between attitudes towards nursing occupation and students’ intentions to pursue nursing education. Students who supported that jobs had a supply and demand balance (0.61%), nurses’ career prospects were very optimistic (2.57%), the work environment was very friendly (2.04%), social status was very high (2.07%) and sense of achievement was very high (1.84%) were more likely to choose nursing specialty than anyone else. The multivariate analyses revealed a significant association between perceived employment situation for nurses and students’ intentions to pursue nursing education (OR = 0.76, *p* = 0.02). Opinions on career prospects (OR = 1.40, *p* = 0.04) and work environment (OR = 1.43, *p* = 0.02) were significant predictors of students’ intentions to pursue nursing education in the univariate analyses. Similarly, nurses’ social status (OR = 0.56, *p* = 0.01), associating nursing with a sense of achievement (OR = 1.51, *p* = 0.048) and knowledge of nursing roles (OR = 1.76, *p* = 0.001) were significant predictors of participants’ intention to study nursing in multivariate analyses (Table 3).

### The impact of the COVID-19 on the nursing profession

The study also investigated the role of the epidemic in influencing participants’ nursing specialty preferences (Table 4). Over 80% (83.65%) of the students thought that nurses played an important role in the epidemic. Approximately 40.7% of students described that the epidemic increased their active understanding of nursing, and 1.28% of them expressed interest in studying nursing compared to 0.36% of those who did not (OR = 0.22, *p* < 0.01). Furthermore, 2401 students (29.82%) felt that the epidemic had a positive impact on applying for nursing, and only 348 students thought the impact was negative. Students with positive views were more likely to choose a nursing major (OR = 0.52, *p* < 0.01).

**Table 4:** The impact of the COVID-19 on the nursing profession

Item	N	N^*^(%)	Univariate regression		Multivariate regression	
			OR (95% CI)	P value	OR (95% CI)	P value
**Role of nursing staff in the epidemic**			1.04(0.77, 1.40)	0.80	0.90(0.66, 1.22)	0.49
Extremely unimportant	122	1(0.82)				
Unimportant	234	5(2.14)				
Average	960	6(0.63)				
Important	2991	12(0.4)				
Very important	3743	35(0.94)				
**Increase active understanding since the outbreak**			0.22(0.18, 0.27)	< 0.01	0.47(0.25, 0.85)	0.01
Yes	3273	42(1.28)				
No	4777	17(0.36)				
**Impact of the epidemic on applying for nursing**			0.52(0.43, 0.53)	< 0.01	1.33(1.00, 1.77)	0.049
Mostly positive effects	2401	23(0.96)				
Mostly negative effects	348	1(0.29)				
No significant effects	5301	35(0.66)				

Multivariate regression models highlighted the significant role of the pandemic in increasing participants’ understanding of various concepts in nursing (OR = 0.47, *p* = 0.01) and emphasized that the impact of the epidemic on major choice was a significant predictor of participants’ intentions to pursue nursing education (OR = 1.33, *p* = 0.049). (Table 4)

## Discussion

The shortage of nurses has threatened the development of the health care system for a long time. Starting with the root of the nursing shortage, the current study aimed to explore senior high school students’ intentions to choose nursing majors in China. According to the study, only 0.73% of the participants had a clear intention to study nursing, which is apparently lower than the proportion of students choosing clinical medicine [17, 18]. This result may be related to the public’s traditional impression of nursing work. Young people seeking to study medicine believed that they would obtain greater power and status as doctors than as nurses [19]. In addition, nursing is less attractive to high school students than other majors, such as economics or computer science. Many factors have contributed to this phenomenon, for instance, the distorted image of nursing, lack of full social recognition, less attractive income and increasing doctor‒patient conflicts [20]. These results were similar to those of our research.

The feminine image of a “nurse” has long been a barrier for men considering nursing as a career [21]. Male nurses play a major role in emergency, intensive care and technical or high-acuity areas [22]. In contrast to previous findings, gender was not a significant predictor for the choice of nursing in our research [23]. It seems that gender stereotypes have gradually been broken, and with the development of the modern nursing industry, the role and functional scope of nursing staff continue to expand. Compared with female nurses, male nurses have more advantages in physical strength, logical thinking, and the skills in using equipment, and the role of male nurses in nursing work is increasingly valued, which is conducive to the development of the nursing industry. The performance ranking was significantly associated with nursing specialty choice. Poor performers were more likely to study nursing, which was due to the lower academic qualifications required for nursing [24]. However, such an association between low academic achievement and increased preference for nursing may hinder the development of high-quality nurses. With the development of the nursing discipline, the traditional nursing model can no longer meet current medical needs. Nurses need to be more professional and have independent dialectical thinking ability. Under the guidance of nursing service guidelines and technical standards, some nursing posts, such as specialist nurses and case managers, have emerged in China in recent years. Statistics in 2017 showed that more than 900 specialized nursing clinics have been opened in China [25], and the scope of nursing practice and service fields has been expanded. To date, more than 70 countries and regions around the world have established the role of senior practice nurses [8], of which 44 countries or regions have granted limited prescription rights to advanced practice nurses (APNs) [26]. In response to the rapid development of the nursing profession, more students with excellent academic performance are required to join the team.

Similar to our findings, studies among Asian participants have reported a significant association between parental influence and students’ consideration for nursing careers [27]. High family support predicted a greater possibility of preferring nursing education among the participants. This could be explained by a highly collectivistic Chinese culture, where young people’s career choices are mainly affected by honouring parental and societal expectations and parental requirements to follow a prescribed career path [28]. Moreover, students with career choices supported by parents receive more emotional and instrumental involvement for their personal growth, resulting in good career adaptability and effective career decision-making self-efficacy [29]. Students who perceive more career support from their parents will gain more vocational identity, may tend to invest more time and passion in pursuing their choice and are likely to engage in more in-depth exploration of their career [30]. Based on these findings, educational institutions should cooperate more closely and actively with parents, give full play to the role of parents in students’ career choice and help students choose nursing majors. Clear guidelines and practical suggestions should be provided to encourage parents to provide career-related support. Examples of this include oral encouragement, emotional support, advice and information, and instrumental help. All parents’ support will help students reduce their self-doubt and commit to career paths, thus enhancing their career adaptability in nursing.

Personal interest in a career has been an important factor in choosing a major [31]. Consistent with our findings, students who were interested in nursing were more likely to choose a nursing major. Cultivating students’ interest in nursing might be a good way to solve the nursing shortage. For instance, in the United States, universities and nursing schools offer various training and learning opportunities, such as free online courses, to stimulate students’ interest and encourage them to consider pursuing a career in nursing [26]. We found that students’ high frequency of receiving nursing-related information and more participation in nursing-related activities were positive predictors of students’ intention to study nursing. Therefore, it is necessary to establish and improve the way to learn about nursing-related information. Fictional medical television programs have been proven to motivate students to enter nursing because they help students make sense of the world of patient care that they are about to enter [32]. Other steps, such as standardizing campus information and offering career planning courses, should be taken to promote more people’s access to nursing-related information. In addition, to promote exposure to health care-related work, activities such as medical science popularization, medical lectures, nursing experience courses and voluntary work in health care settings should be held.

In accordance with our findings, career prospects were a significant predictor of senior high school students’ preference for nursing education [33]. This might be attributed to the fact that nursing offers better employment opportunities and greater career mobility. In China, the current nursing shortage promises job security to nursing students. The Chinese government has developed various measures to attract, train, and retain recent nursing graduates [34]. The role of nurses could be extended to advanced nurse practitioners, researchers, educators, and managers. China is vigorously developing the construction of nursing disciplines and further improving the model of high-quality, specialized and compound nursing talents. According to the “National Nursing Development Plan (2021–2025)”, a nurse training system guided by job requirements and centred-job competence should be established. Position-based and classified measures should be adhered to in order to effectively improve the clinical nursing service ability of nurses. This study provides guidance for nurse promotion and nursing career planning and provides a basis for good prospects for nursing careers.

Participants in the study had mixed views on nurses’ social status, partly explaining the variation in the preference for nursing education. Social status has become the main reason why students from low- to middle-income countries and upper- to middle-income countries choose medicine [35]. However, only 38% of the participants in this study associated nursing with high social status. Nurses often spend a considerable amount of time providing care to patients but are regarded as service personnel [36]. Currently, the clinical treatment work of nurses is mostly subject to doctors in China, which inevitably leaves the public with the replaceability, repeatability and mechanical characteristics of nursing work. Lack of authority and autonomy has long been an obstacle preventing nursing from standing out as a full career in China [37]. There is a need for nursing scholars to explore the boundary of nursing responsibilities and ways to gain authority and autonomy for nursing and ultimately improve nursing status. We observed that students who supported a very high sense of achievement were more likely to choose nursing specialties. A heightened sense of achievement among nurses could be classified as altruism, which is a significant motivation for pursuing nursing education and contributes to job satisfaction and to nurses’ moral competency [38, 39]. Thus, it is necessary to consider how to increase the attractiveness of altruistic values and promote the unique characteristics of the nursing profession. Sharing nurses’ personal stories of altruism and dedication may be a considerable way to do so. Furthermore, the value, dignity and reward of nursing work should be emphasized, and the value of the nature of work should be linked with the sense of work achievement [40].

Some students expressed that the epidemic increased their understanding of nursing and had positive effects on nursing application in the present study. Personal epidemic experience and continuous media reports could have increased students’ attention to nursing. Many people recognize that nurses are the largest group of health care professionals at the forefront of the medical team, providing medical, emotional, and psychological care to patients. On the other hand, nurses have been labelled “heroes” by politicians, the mass media, and the general public [41]. All these factors improve the public awareness of nurses’ social status and advocate altruistic characteristics, ultimately forming positive effects on nursing application. However, the image of heroism hides daily, mundane, dirty and gendered nursing work [42]. The praise and enthusiasm for nursing during COVID-19 may quickly fade away and may not result in profound impacts on the recruitment of nursing students according to the same phenomenon that occurred during the 2003 severe acute respiratory syndrome outbreak [43]. Politicians and policy-makers should turn their appreciation of nurses into positive actions. What they need to do is actively address some long-term issues faced by nurses, such as low wages, heavy workload and low status within the health care power hierarchy. The International Council of Nurses recommended that health care employers and organizations should provide better working conditions and more support for nurses in the postepidemic era.

The current study had numerous limitations. First, the study drew participants from 5 senior high schools in five provinces of China. The study sample may not accurately reflect the entire population. Future studies should focus on more high school students across a more comprehensive geographic range to enhance the generalization of findings. Second, the questionnaire used in this study is a self-assessment questionnaire rather than a scale, so it could not be strictly tested for validity. Although the validity of this questionnaire had certain limitations, it still may reflect some problems to some extent. Last, the study’s cross-sectional nature limits the consideration of the association between the relevant covariates and students’ preferences for nursing education over time. Longitudinal studies are necessary to infer the effect of changing perceptions on nursing education preference.

## Conclusion

According to the study, only a small proportion of high school students intend to pursue nursing education. Academic performance and family support are significant predictors of students’ intentions to pursue nursing education. With the deepening understanding of the nursing specialty, a more positive cognition of nursing occupation and the experience of the COVID-19 pandemic, students are more likely to choose a nursing career. This study hopes to provide a new perspective for predictors of nursing shortages and potential interventions. Relevant stakeholders should implement various strategies to enhance the consideration for nursing education and careers among high school students.

### Electronic supplementary material

Below is the link to the electronic supplementary material.

Supplementary Material 1

## Data Availability

The datasets used and/or analysed during the current study are available from the corresponding author on reasonable request.

## Abbreviations

APN: Advanced practice nurse
ICN: International Council for Nurses
ANA: American Nurse Association
