# Supplementary material for: Investigating the intentions and reasons of senior high school students in registering for nursing education in China
Source: BMC Nurs. 2023 Sep 12;22:311. doi: 10.1186/s12912-023-01480-w (PMC10496206; doi:10.1186/s12912-023-01480-w)
Supplement: Supplementary file 1 — Supplementary Material 1 [file 12912_2023_1480_MOESM1_ESM.docx]

# **Questionnaire**

# **An investigation of the intention and reasons of senior high school students in China to register for nursing**

Dear participants,

We are going to conduct a survey on the intention of senior high school students to register nursing in China and its influencing factors. Your specific situation meets the inclusion criteria for this study. We invite you to participate in this study. Your participation in this study is voluntary and has been reviewed by the Ethics Review Committee of Shanghai General Hospital affiliated with Shanghai Jiao Tong University School of Medicine. The content of the questionnaire is centered on your intention to apply for the nursing profession. At the same time, the questionnaire will mainly focus on your personal and family information, your understanding of the current nursing education model, the way you obtain the nursing specialty information, your cognition of the nursing profession or image, and your view on the nursing profession after experience the COVID-19 . The estimated filling time is 10 minutes. The questionnaire will be distributed by members of the research team and collected on site after completion.

Thank you very much for taking the time out of your busy schedule to complete this questionnaire. Your information will be an important source of data for this study. Please choose the most suitable option for each question based on your own real situation and thoughts. There are no standard answers to these questions.

1. Gender

□ Male

□ Female

1. Grades

□ Senior one

□ Senior two

□ Senior three

3.What’s your academic performance ranking usually?

□ Top 5%

□ 6%~ 30%

□ 31%~70%

□ 71%~94%

□ Bottom 5%

4.What courses do you choose?

□ Liberal Arts (Partial Liberal Arts)

□ Science (Partial Science)

5.What is monthly income of your family?（RMB per person）

□ <2000

□ ≥2000 and <5000

□ ≥5000 and <10000

□ ≥10000 and <20000

□ ≥20000

1. Is one or both of your parents medical professionals?

□ Yes

□ No

7. What is the main attitude of your family members or acquaintances towards applying for the following medical majors?

□Oppose

□Neutral

□Support

8. Are you interested in becoming a nurse？

□ Totally uninterested

□ Uninterested

□ Neutral

□ Interested

□Very interested

9. How do you pick up information about the nursing profession?

□ I know nothing about education of the nursing profession

□ Traditional media(e.g. book)

□ New media(e.g. network, app)

□ School lecture

□ Heard from people around

10. How often do you receive information about the nursing profession?

□ Never

□ Extremely low

□ Low

□ High

□ Extremely high

11. Have you ever participated in any medical practice or popular science activities?

□ Yes

□ No

12.What do you think of the current employment situation for nurses?

□ Job supply far greater than demand

□ Job supply and demand balance

□ Job supply far less than demand

13. What’s your opinion about nurse’s career prospects?

□ Very pessimistic

□ Pessimistic

□ Neutral

□ Optimistic

□ Very optimistic

14. How friendly is the current medical work environment for nurses?

□ Extremely unfriendly

□ Unfriendly

□ Average

□ Friendly

□ Very friendly

15. What’s your opinion about nurse’s social status?

□ Very low

□ Low

□ Average

□ High

□ Very high

16. What’s your opinion about Nursing is a job with great sense of achievement?

□ Very low

□ Low

□ Average

□ High

□ Very high

17.Do you think you know a lot about the work of nurse?

□ Strongly disagree

□ Disagree

□ Neutral

□ Agree

□ Strongly agree

18.What is the attitude of the nurses you come into contact with towards patients?

□ Very poor

□ Poor

□ Fair

□ Good

□ Very good

19.Your current intention to enrol in the nursing programme is

□ Very low

□ Low

□ Average

□ High

□ Very high

20.Have you ever experienced a confirmed diagnosis or 14-day autonomous medical quarantine?

□ Yes, I have been a confirmed case

□ Yes, I have experienced isolation

□ No, but someone I know well has

□ No, neither I nor anyone around me has experienced it

21.Are there family members who became health care workers on the front lines of the fight against the epidemic?

□ Yes

□ No

22. The role of nursing staff in the epidemic is important

□ Strongly agree

□ Agree

□ Neutral

□ disagree

□ Strongly disagree

23. Have you increased your active understanding of the nursing profession since the outbreak of the epidemic?

□ Yes

□ No

24. The impact of the epidemic on your willingness to apply for nursing is____

□ Mostly positive effects

□ Mostly negative effects

□ No significant effects
